# There is not one public: divergent perceptions of scientists’ source credibility in the United States

**DOI:** 10.1057/s41599-026-07609-x

**Published:** 2026-07-02

**Authors:** Shumaila J. Bhatti, Dara M. Wald

**Affiliations:** 1 School of Public and International Affairs, Virginia Tech, Blacksburg, Virginia US; 2 Fralin Life Sciences, Virginia Tech, Blacksburg, Virginia US

**Keywords:** Psychology, Psychology, Science, technology and society

## Abstract

The public relies on source credibility as a cognitive shortcut when deciding whether to accept or reject science-informed policies. Using data from a subsample of a nationally representative U.S. survey (*N* = 927), we applied Latent Class Analysis to identify five distinct audience segments based on their perceptions of scientists’ source credibility, defined as competence, integrity, and goodwill. The LCA was replicated across two environmental health contexts: extreme heat and a zoonotic disease associated with foodborne illness. Multiple regression models were also estimated to determine how individuals’ characteristics, such as a priori attitudes about science and scientific institutions, attention to media and interpersonal sources, general social trust/distrust, and sociodemographic variables were associated with perceived scientific source credibility and policy support across both contexts. Enthusiasts were characterized by exceptionally high credibility perceptions, institutional confidence, and policy support with Optimists having similarly high, though context-dependent responses. Moderates had intermediate levels of perceived scientific source credibility and exhibited balanced information-seeking patterns, relying on both technical and personal sources. Skeptics and Estranged groups exhibited lower credibility perceptions and institutional trust than other groups, with the Estranged representing the most disconnected segment. Perceptions of scientific competence were higher across all the segments compared to integrity and goodwill. Regressions revealed strong associations between source credibility, perceived scientific motivations, and institutional confidence in scientists. Class assignments were also strongly associated with optimistic beliefs about science and support for science-informed policies. By recognizing these segments and their context-dependent concerns, scientists can tailor messages to support evidence-informed policymaking.

## Introduction

Credibility, rooted in Aristotle’s concept of ethos (intelligence, character, and goodwill), alongside logos (logical reasoning) and pathos (emotional appeal), is a multidimensional construct central to persuasive communication (Aristotle, 1991; Heath, 2013; McCroskey & Teven, 1999). Scholars have proposed multiple names for the components of source credibility, including competence, honesty, concern, fairness, and trust (Besley et al., 2021; Cologna et al., 2025; Horton et al., 2017; Hunt & Wald, 2020; Kotcher et al., 2017; McComas et al., 2014; McCroskey & Young, 1981). Despite debate about naming, contemporary research has generally validated Aristotle’s framework, identifying three key elements of source credibility: competence (e.g., expertise, intelligence, qualification); integrity (e.g., honesty, trustworthiness, and openness), and goodwill or benevolent/caring intentions toward the receiver (Hovland et al., 1953; Hunt & Wald, 2020; McCroskey & Teven, 1999; McCroskey & Young, 1981).

The public uses perceptions of source credibility as a cognitive shortcut to interpret scientific messages (Hovland & Weiss, 1951, 1952; Li & See-To, 2023; Pornpitakpan, 2004). Audiences skeptical of scientists’ credibility are more likely to ignore their recommendations and reject their messages (Nisbet et al., 2015; Pechar et al., 2018; Vraga et al., 2018), making skepticism about scientific credibility a significant barrier to effective science communication. Indeed, source credibility has been identified as a critical factor shaping public opinions of science (Brossard & Nisbet, 2007), scientific messages (Pornpitakpan, 2004), and the legitimacy of scientists to inform science policy (O’Brien, 2013; Wald & Williams, 2017). When scientific findings are controversial or complex, such as in the case of anthropogenic climate change, media effects on audience beliefs are regulated by audiences’ perceptions of scientific source credibility (Krosnick et al., 2006).

Early scholarship on source credibility established that receiver characteristics, including a priori beliefs and sociodemographic variables (e.g., knowledge, gender, religious or political identification), shape perceptions of source credibility. For example, individuals exhibit more positive perceptions of scientists when recommended policies align with their personal views (Cologna et al., 2022; Pornpitakpan, 2004). This is referred to as ‘biased assimilation,’ defined as the tendency for individuals to accept supportive information uncritically while scrutinizing opposing evidence leading to increased polarization (Lord et al., 1979). Previous scholarship shows that public perceptions of scientific source credibility can also be polarized (Hunt & Wald, 2020) and shape audience responses to scientific messages (Li & See-To, 2023). Ample scholarship suggests that perceptions of source credibility vary among individuals and groups, as a socially and culturally constructed phenomenon (Peters et al., 1997; Fiske & Dupree, 2014; Hamilton et al., 2015). More recently, Horton et al. (2025) posit that source credibility is relational and situational; therefore, in some cases, and to some audiences, scientists’ ability to demonstrate their integrity or openness may be more important than their competence (as demonstrated by their advanced degree or awards). Yet few empirical studies to date have examined how the different elements of source credibility may vary and operate among different audiences and across specific contexts.

Considerable scholarly effort has previously focused on receiver beliefs and cognitive processes to identify audience segments. Audience segmentation enables researchers to uncover latent patterns in how different groups evaluate and respond to information sources, with applications in climate change (Maibach et al., 2009), science (Besley, 2018; Schäfer et al., 2018), and health (Richardson et al., 2023; Smith, 2017) revealing distinct audience characteristics, beliefs, attitudes, and behaviors. One of the more well-known examples of this is the Six Americas framework proposed by the Yale Climate Change Communication Center that has been applied to other countries as well such as the five Canadas (Chryst et al., 2018; Martel-Morin & Lachapelle, 2022). A nationally representative study from 2002 to 2010 on the US public’s support for embryonic stem cell research identified four distinct audience segments: Scientific Optimists (highly educated, financially secure, predominantly white individuals strongly supporting scientific progress), Scientific Pessimists (lower educational attainment and income individuals with moral reservations about biomedical advances), the Conflicted (socially similar to Pessimists but older and ambivalent, yet open to benefit-focused arguments), and the Disengaged (less informed individuals lacking well-formed mental models who rely on partisan cues and focusing events for opinion formation) (Nisbet & Markowitz, 2014). Most segmentation studies group audiences by beliefs and values, not by source perceptions. This approach is grounded in the assumption that personal values supersede source effects in influence – despite limited empirical examination of these factors in tandem (Li & See-To, 2023; Otis, 2024). Even as decades of persuasion scholarship have foregrounded message and receiver properties, environmental and science communication research has to date, insufficiently integrated source perceptions into audience segmentation frameworks (Pornpitakpan, 2004). This research addresses these gaps by examining how and whether different subgroups exist due to perceived scientific source credibility and how they may vary across contexts.

### Literature review

Scientific “truths” are embedded in and informed by individual and social realities, including politics, religion, economics, and culture (Jasanoff & Kim, 2013). Source credibility is also informed by political ideology, culture, and context (Horton et al., 2016; Hunt & Wald, 2020). For example, a study of public perceptions of scientific competence, benevolence and integrity across 68 countries revealed significant differences across locations, influenced by political, cultural, and socioeconomic factors (Cologna et al., 2025). Broadly, findings revealed greater reported trust in scientists across Western Europe, Nordic countries, and East Asia, compared to respondents from Russia, parts of Eastern Europe, and some Latin American and African countries (Cologna et al., 2025). In addition to cultural differences, credibility may also operate differently across contexts. Besley et al. (2021) reported that the association between perceived source competence and public support for scientific research varied depending on whether the research in question was on GM technology, nuclear energy, climate change or autonomous vehicles. While the authors suggested it is possible that general perceptions of scientists may not predict support for specific types of research, it is also possible that this finding highlights the contextual, cultural, and situational nature of credibility. Indeed, as work by Besley & Tiffany (2023) points out, “peoples’ perceptions of experts and their willingness to trust experts’ guidance as legitimate is socially constructed around specific subjects” (pg 724). Horton et al. (2025) further posits that credibility is fluid and co-constructed within each communicative context. For example, perceptions of source credibility were more effective than message appeal in electronic word of mouth (eWOM) responses in social media environments using the Elaboration Likelihood Model (ELM) compared to message appeal (Li & See-To, 2023).

Previous scholarship has established that communication about controversial scientific issues can influence perceptions of a source’s political orientation (Vraga et al., 2018). Other research has reported that the relationship between solution position preference and perceived credibility is mediated by perceptions of scientists’ motivations, particularly motivations to serve and persuade the public (Beall et al., 2017). Drawing on this body of knowledge, we posit that scientific source credibility is a perceptual, relational construct that emerges from some combination of competence, integrity, and goodwill (Aristotle, 1991; Horton et al., 2016; Horton et al. 2025; Hunt & Wald, 2020). Therefore, for some audiences and across contexts with differing levels of controversy, credibility as goodwill may be more salient than credibility as competence. To assess this, we ask two research questions:

(RQ1) How many subgroups are there when it comes to public perceptions of scientific source credibility in the U.S.?

(RQ2) How do the different dimensions of scientific source credibility and its antecedents vary across subgroups and in controversial and non-controversial contexts?

Early scholarship on source credibility established that receivers’ characteristics can influence a person’s perceptions of a source and their message (Pornpitakpan, 2004). Individuals’ perceptions of scientists are in turn, associated with their attitudes toward controversial scientific topics such as cell research (Liu & Priest, 2009), climate change (Hmielowski et al., 2014) or nanotechnology (Vandermoere et al. 2010). Subsequent research has identified several key antecedents of perceived source credibility including a receiver’s attention to media and interpersonal sources (Brondi et al., 2025; Nisbet et al., 2015). Social representation theory asserts that mass media exposure fundamentally shapes the formation of one’s beliefs about science (Brondi et al., 2025; Van Lange et al., 2011). The theory generally asserts that responses to broadcast media are shaped in part by exposure to social debate and discussion within one’s social network (Hwang & Southwell, 2009). Scholars who take this view do acknowledge that mass media has a unique influence on perceptions of science. And some research has reported evidence of a mass media effect. For example, Nisbet and Lewenstein (2002) found that exposure to science- or nature-dedicated programming reduced reservations about science, while general programming exhibited somewhat negative effects on science-related beliefs. Similarly, Besley and Shanahan (2005) reported that attention to television science news positively predicted support for agricultural biotechnology. But social representation theory also highlights the importance that interpersonal conversation plays alongside mass media exposure in shaping individual beliefs and social discourse (Bauer, 2006; Bauer & Gaskell, 1999; Stewart & Lacassagne, 2005). This line of scholarship suggests that participatory behavior because of media exposure is largely dependent on whether individuals also discuss politics with other people (Scheufele, 2002). Despite evidence linking information source seeking through technical (science-specific media) and personal (interpersonal conversation) sources to science attitudes and risk perceptions, a critical gap persists in understanding how these information-seeking behaviors relate to source credibility perceptions. Thus, this research will add to current understanding of audiences’ attention to science-specific media and interpersonal sources and how these may shape perceived scientific source credibility.

Scientific institutions are generally contextualized as governing ‘factual’ bodies of scientific knowledge (Sturgis & Allum, 2004). Thus, in many cases, beliefs about expert sources are also mediated through knowledge-producing institutions that may be associated with specific scientists or scientific research (Sturgis & Allum, 2004). Some knowledge of scientific institutions can help to contextualize and ground what individuals accept as credible and legitimate. Indeed, greater familiarity with scientific research is associated with strong agreement that scientists provide fair and accurate information, are competent, and show concern for the public (Funk et al., 2019b). In the context of environmental resource management, confidence in the credibility of specific scientists or technical experts is often higher than perceived organizational credibility (e.g., resource agencies) (Wald et al., 2019). Yet in a recent meta-analysis, Cologna & Siegrist (2020) suggest that the potential association between attitudes toward scientific institutions and attitudes toward specific scientists is dependent on value similarity between the person choosing to trust and the institutions/organizations being considered. Therefore, it is possible that perceived source credibility is influenced by complex interactions between individual attitudes toward science and beliefs about scientific institutions. We include both measures of confidence in scientists and institutions in this study.

Positive relationships with expert sources and confidence in their competence, integrity and goodwill has also been associated with one’s level of confidence (the belief, based on experience, that experts will act as expected) and social trust (based on value similarity) (Earle, 2012). Besley et al. (2021) found strong (>.80), significant associations between perceived scientific integrity, benevolence and openness and a generalized measure of trust. A COVID-19 vaccination study found that low social trust weakened the link between message usefulness and vaccination intent, showing trust’s key role in turning source credibility into acceptance (Maduku, 2024). Conversely, in more recent scholarship, social trust had a weak association with environmental behavior (Cologna & Siegrist, 2020). In this study, we include a measure of general social trust in this study as a potential antecedent of perceived scientific source credibility.

In the U.S., one’s self-identified position on the liberal-conservative scale is consistently associated with polarized responses to environmental issues, including climate change, nuclear power, and GM foods (Funk et al., 2019a; Hamilton et al., 2015; Nisbet et al., 2015). In addition, public opinion about scientists and researchers is, in many cases, divided along political lines (Funk et al., 2019a; Gauchat et al., 2017; Gligorić et al., 2025; Hunt & Wald, 2020; McCright, 2016; Vraga et al., 2018). Perceptions of environmental health specialists and environmental researchers, which are the topics included in this study, were among the most politically polarized in US-wide surveys of the public (Funk et al., 2019a). Studies have found that those leaning left in political ideology, women, and younger audiences held more positive views of scientists and confidence in environmental scientists’ understanding of climate change (Besley, 2015; Cologna et al., 2022; Gauchat et al., 2017). Among certain audiences, such as Republican and Evangelicals, scientists and their knowledge systems have been perceived as a social threat, leading to support for excluding scientists from policy-making and, in some cases, retributive actions against scientists. (Chinn et al., 2024). Polarization extends to perceptions of scientists’ credibility as well, with conservatives in the U.S. substantially more skeptical of scientific (and scientists’) competence, integrity, and goodwill (Gauchat, 2012; Gauchat et al., 2017; Hunt & Wald, 2020a; Hunt & Wald, 2018). Further, when communicating about controversial scientific issues, audience members make inferences about scientists’ political affiliations and these interpretations can shape audience perceptions of scientific credibility (Kotcher et al., 2017; Vraga et al., 2018).

Perceptions of scientists have also been associated with sociodemographic variables (e.g., age, gender, education). Higher scientific knowledge, technical skills, and advanced degrees have been associated positively with assessments of scientific credibility (Hayat et al., 2019; Metzger & Flanigan, 2015). However, Besley et al. (2021) argues that these findings reflect experiences with scientists rather than a direct causal relationship between demographics and perceived source credibility. For example, individuals with technical skills are more likely to have advanced degrees or direct experiences with scientists and be more connected to scientific institutions, which may contribute to greater positive perceptions of scientific source credibility (Gauchat, 2011). Nevertheless, we included these socio-demographic variables, as others have done, to further examine the multitude of the factors that may interact and contribute to public perceptions of scientific source across contexts.

This body of work suggests that scientific source credibility is not a fixed quality but emerges in varied ways shaped by receivers’ characteristics. One additional factor less examined in the literature, but included here, is scientists’ perceived motivation. The Pew Center’s Politics of Climate report in 2016 found that only 23% of U.S. adults believed that climate scientists’ research findings were “influenced by concern for the best interests of the public” most of the time and only 32% believed they were “influenced by the best available science” most of the time (Funk & Kennedy, 2016). In other words, most Americans were skeptical of climate scientists’ motivations. This has important implications for science policy because previous research suggests that public support for scientific influence on policy increases when scientists are perceived as motivated by public interests (Árvai et al., 2020; O’Brien, 2013). Drawing on small-group interviews, Wald et al. (2019) reported that skepticism about decision-makers’ motivation, and particularly their focus on achieving the goals of their own research, rather than a specific environmental outcome, reduced support for environmental management. Bonnie et al. (2020) found that one of the primary sources of skepticism about environmental policies among rural audiences is the perception that rural voters’ interests are excluded from policy discussions and environmental recommendations. As one forestry leader put it, “Whether you’re Republican or Democrat, if you serve in office, it’s for you and not for the people” (Bonnie et al., 2020). Drawing on these studies, we posit that public perceptions of scientists’ motivation are associated with perceived scientific source credibility and may also shape support for specific science-informed policies.

There is also a substantial body of work connecting beliefs about scientists and scientific institutions, including perceived integrity and competence to support for science-informed policies. Early work in the field suggests that source credibility enhances the effectiveness of communication about science-informed policy and action (Gauchat et al., 2017; Hovland & Weiss, 1951, 1952; Pornpitakpan, 2004; Teven, 2008). Besley et al. (2021) suggested that one’s perception of the benefits of science may mediate the relationship between perceived scientific integrity and support for scientific research. Kang and colleagues found that individuals with optimistic beliefs about science and technology had high levels of environmental concern, liberal political ideology, and expressed positive support for gene drive policy, viewing scientific advancement as beneficial for addressing environmental and societal challenges (Kang et al., 2023). Vandermoere et al. (2010) posited that individuals with positive beliefs about science are more likely to seek positive scientific information from the media and discuss science with close contacts, which in turn, contributes to positive attitudes toward a specific scientific topic or those who conduct research in this field, though they did not ask participants about information seeking behaviors.

Given this body of work, the inclusion of a beliefs measure is critical for understanding how audience-level difference in perceptions of scientists’ credibility shape engagement with science-informed policy. Prior research indicates that credibility perceptions may lead to positive belief spillovers across health and environmental contexts (Bancale et al., 2024), suggesting that individuals who hold favorable beliefs about scientific integrity may generalize this trust to multiple areas of science-informed behavior. Examining beliefs reveals how perceived credibility drives policy support and belief spillover across domains. Yet these links remain unclear across audiences and contexts. This study aims to clarify how beliefs about science and source credibility shape public support for science-based action across environmental health issues. We therefore ask:

(RQ3) How does confidence in individual scientists and scientific institutions, attention to sources, general social trust, political and sociodemographic factors, and beliefs about scientists’ motivation influence perceptions of scientists’ source credibility across varied contexts?

(RQ4) How does support for environmental action and vaccination vary across subgroups and contexts?

## Methods

### Experimental design

We recruited a representative sample of the U.S. population (*N* = 3113) through Qualtrics between December 12, 2023, and January 9, 2024. Prior to starting the survey, all participants gave informed consent. The consent statement explained that the study examined perceptions of science, scientific beliefs, and scientists. Participants confirmed they were at least 18 years old and understood that their participation was voluntary, their data would be de-identified, and they could withdraw at any time. Consent was recorded when participants clicked “next” to begin the survey. Qualtrics Panel Services compensated respondents for their participation. The Human Research Protection Program at Texas A&M (IRB2023-0827) approved the study protocol. To ensure data integrity and minimize response bias, multiple quality control measures were implemented (Perfecto & O’Donnell, 2025). These measures included embedded attention checks throughout the survey, automated bot detection algorithms, and recording of click-through rates on presented news articles. Attention checks comprised of reverse-coded items designed to identify inconsistent or careless responding patterns. Only responses that met completion criteria across all survey sections were retained for subsequent analysis after bot-like responses were identified and removed by Qualtrics.

This study was part of a larger study that included experimental manipulation. Briefly, the experiment employed a 2 (topic: controversial vs. non-controversial) × 2 (language: hedging vs. no hedging) × 3 (source credibility: no signals vs. integrity vs. goodwill) factorial design. Participants read a news article before completing the survey. We developed the articles specifically for this study based on previous Associated Press reports, focusing on the impacts of extreme heat (the controversial topic) and Salmonella outbreaks (the non-controversial topic) in the Southern United States. Each article ranged from 260 to 300 words. Studies from media effects also demonstrate that strategic news frames generate greater cynicism and distrust compared to issue-based frames, with issue-based news eliciting more public comments and reactions (Stroud & Muddiman, 2019). We designed the stories to be nonpolitical by emphasizing health and economic impacts (issue-based) rather than political dimensions (strategic frames). In the study, a key manipulation was to test the effects of scientists communicating uncertainty. We did this by creating news articles using hedging. Hedging, a linguistic device that marks verbal (lexical or discursive) or numerical (range) ambiguity or uncertainty (Hyland, 1996, 2017), represents one method of communicating uncertainty in scientific communication. To create the hedging manipulation, we developed two versions of each article in which hedging words comprised either 8–10% of the total text (hedging condition) or were absent (no hedging condition). We replaced the names and identifiers of specific health officials and legal agency representatives with common nouns to maintain generalizability.

### Sample selection

We conducted preliminary analyses using MANOVA to examine the effects of hedging perceptions of scientists’ competence, integrity, and goodwill. Results indicated no significant effect of hedging on any dimension for either topic condition. For the heatwave condition: competence (Pillai’s Trace = 0.008, F(1, 13) = 0.871, *p* = 0.58), integrity (Pillai’s Trace = 0.006, F(1, 13) = 0.597, *p* = 0.86), and goodwill (Pillai’s Trace = 0.006, F(1, 11) = 0.749, *p* = 0.69). For the Salmonella condition: competence (Pillai’s Trace = 0.006, F(1, 6) = 1.300, *p* = 0.25), integrity (Pillai’s Trace = 0.008, F(1, 13) = 0.871, *p* = 0.58), and goodwill (Pillai’s Trace = 0.01, F(1, 11) = 1.289, *p* = 0.22).

Based on these findings, we selected participants exposed to hedging and no hedging conditions for the present analysis (*n* = 927). We excluded participants who were exposed to the credibility signals, as MANOVA revealed significant effects of these signals on outcome variables, and testing effects of messages on audience segmentation is currently outside the scope of this study. This exclusion ensured that message effects did not influence latent class assignments. We also excluded participants with incomplete sociodemographic data. For example, due to the small number of non-binary and transgender participants (*n* = 6) in the overall sample, we restricted the analysis to participants who identified as man or woman.

### Sample characteristics

The final sample consisted of 927 participants randomly assigned to either the heatwave condition (*n* = 466) or the *Salmonella* condition (*n* = 461), with mean ages of 54 years (range: 18–95) and 56 years (range: 18–93), respectively. The sample was evenly split by gender (49.4% and 49.0% men, respectively), predominantly non-Hispanic (45.6% and 46.4%), and majority White/Caucasian (39.1% and 39.6%), with representation from Black/African American (7.2% and 5.9%) and other racial groups (4% and 4.2%). Political affiliation was distributed across Democrats (20.3% and 20.1%), Independents (17.6% and 18.2%), and Republicans (12.4% and 11.4%), with political ideology showing a relatively normal distribution centered on neutral/moderate positions. Participants were geographically distributed across the South (18.7% and 19.1%), Midwest (11.9% and 12.3%), West (10.1% and 10.9%), and Northeast (9.6% and 7.4%), with educational attainment ranging from some high school (1.8% for both conditions) to graduate degrees (5.6% and 6.6%), and the most common level being high school diploma/GED (12.9% and 13.3%). Annual household income was widely distributed, with the largest proportions reporting incomes $100,000 or more (28% and 26.1%), while lower income brackets (below $49,999) were less represented (6.8% and 8.2%). Overall, the sociodemographic categories were approximately evenly distributed across the two topics (heatwave and *salmonella*). The sociodemographic traits of the sample are as follows shown in Table 1.

**Table 1:** Sociodemographic Characteristics (*N* = 927).

Characteristics	Heatwave (*n* = 466)	Salmonella (*n* = 461)
	*N (%)*	*N (%)*
Age *M* (range)	54 (18–95)	56 (18–93)
Gender		
Man	230 (24.8)	226 (24.4)
Woman	236 (25.5)	235 (25.4)
Hispanic		
No	423 (45.6)	430 (46.4)
Yes	43 (4.6)	31 (3.3)
Race		
Black/African American	67 (7.2)	55 (5.9)
Other Races (Asian, Native Americans, etc.)	37 (4)	39 (4.2)
White/Caucasian	362 (39.1)	367 (39.6)
Political Party		
Democrat	188 (20.3)	186 (20.1)
Independent	163 (17.6)	169 (18.2)
Republican	115 (12.4)	106 (11.4)
Political Ideology		
Extremely Liberal	45 (4.9)	43 (4.6)
Moderately Liberal	55 (5.9)	62 (6.7)
Slightly Liberal	45 (4.9)	37 (4)
Central/Neutral	120 (12.9)	118 (12.7)
Slightly Conservative	49 (5.3)	35 (3.8)
Moderately Conservative	82 (8.8)	95 (10.2)
Extremely Conservative	70 (7.6)	71 (7.7)
Region		
Midwest	110 (11.9)	114 (12.3)
Northeast	89 (9.6)	69 (7.4)
South	173 (18.7)	177 (19.1)
West	94 (10.1)	101 (10.9)
Education		
Some high school/less	17 (1.8)	17 (1.8)
High school diploma/GED	120 (12.9)	123 (13.3)
Some college, but no degree	108 (11.7)	95 (10.2)
Associates/technical degree	61 (6.6)	65 (7)
Bachelors degree	108 (11.7)	100 (10.8)
Graduate/professional degree	52 (5.6)	61 (6.6)
Income		
$150,000 or more	110 (11.9)	113 (12.2)
$100,000–$149,999	149 (16.1)	129 (13.9)
$75,000–$99,999	90 (9.7)	87 (9.4)
$50,000–$74,999	54 (5.8)	56 (6)
$25,000–$49,999	36 (3.9)	47 (5.1)
Less than $25,000	27 (2.9)	29 (3.1)

### Measurements

Sociodemographic variables previously linked with source credibility were coded as follows: Gender (1 = man, 2 = woman); education level (1 = less than high school, 2 = high school graduate, 3 = some college, no degree, 4 = Associate’s degree, 5 = college graduate/some postgrad, 6 = graduate/professional degree); age (1 = 18–29, 2 = 30–49, 3 = 50–64, 4 = 65 and older); and income (ranging from 1 = more than $150,000 to 6 = less than $10,000). Political ideology was assessed by having respondents rate themselves on a scale from Extremely Liberal = 1 to Extremely Conservative = 7. Political party affiliation was coded as Democrat = 1, Independent = 2, and Republican = 3.

Participants completed self-reported assessments across multiple domains (perceptions of source credibility, science attitudes, institutional confidence, information seeking sources, social trust, and attitudes toward environmental action and health behaviors), with responses recorded separately for controversial and non-controversial topics. All items used Likert-type scales. Constructs’ internal consistency was validated using confirmatory factor analysis, and reliability was assessed using Cronbach’s alpha (Table 2). The psych package in R was used to apply confirmatory factor analysis with oblique (oblimin) rotation to account for correlations among the items in each of the respective measures (Park et al., 2002; Revelle, 2024).

**Table 2:** Summary of survey item measures.

Measures	Heatwave			Salmonella		
	*M*	SD	*α*	*M*	SD	*α*
Competence	5.38	1.34	0.94	5.69	1.01	0.91
Openness	4.98	1.32	0.97	5.38	1.01	0.96
Benevolence	4.86	1.27	0.92	5.11	0.95	0.87
Science Optimism	5.23	1.14	0.87	5.38	0.90	0.80
Science Skepticism	4.34	1.34	0.81	4.23	1.24	0.78
Environmental Support	4.91	1.59	0.95	4.69	1.63	0.95
Vaccination Support	4.51	1.57	0.94	4.16	1.55	0.94
Technical Information Source	3.08	1.50	0.91	3.02	1.50	0.90
Personal Information Source	2.74	1.52	0.93	2.75	1.58	0.93
Social Trust	4.63	1.25	0.82	4.68	1.24	0.84
Social Distrust	4.77	1.28	0.76	4.72	1.27	0.80
Scientists Influenced	5.23	1.42	0.79	5.55	1.20	0.72
Confidence in General Institutitons	3.87	1.32	0.87	3.92	1.21	0.85
Confidence in Scientists	4.93	1.67		5.26	1.35	

#### Perceptions of source credibility

We used the three dimensions of source credibility: competence, integrity, and goodwill. Perceptions of EH Scientists *Competence* was self-reported using pair-wise items assessing perceptions of scientists working on heatwave- or salmonella-related topics. The final items mirrored the five competence items identified by (Besley et al., 2021) (e.g., “Intelligent,” “Skilled,” “Qualified,” “Knowledgeable,” “Expert”, heatwave *α* = 0.94, salmonella *α* = 0.91). To measure perceptions of EH Scientists *Integrity*, we began with 20 items that were identified by Besley et al. (2021). In Besley’s article, these were identified as a single factor measuring openness, benevolence, and integrity. After removing items that did not exhibit a good fit with the measure, we had 13 pair-wise items including “Honest,” Ethical,” “Character”, “Integrity”, Moral,” “Keeps Promises,” “Improves Lives,” “Wellbeing,” “Dignity,” “Considerate,” “Listens,” “Polite,” and “Welcomes Feedback” (heatwave *α* = 0.97, salmonella *α* = 0.96). Perceptions of EH Scientists *Goodwill*, included nine items developed by modifying previous measures used by Cologna et al. (2022) and Hunt & Wald (2020) such as “Benefits Others,” “Advances Society,” “Creates Accurate Knowledge,” “Draws Independent Conclusions,” “Informs Public,” “Reports Findings,” “Selfless,” “Empathetic,” and “Shares Accurate Information” (heatwave *α* = 0.92, salmonella *α* = 0.87).

#### Confidence

*Confidence in General Institutions* was self-reported with six items assessing confidence in government officials, news media, social media, business leaders, leaders from political party, and leaders from place of worship, with responses ranging from 1 (*no confidence at all*) to 7 (*very confident*; heatwave *α* = 0.87, salmonella *α* = 0.85). *Confidence in Scientists* was self-reported with a single item using the same response scale. These items were adopted and modified from Füchslin et al. (2019) and Kang et al. (2023).

#### Information source seeking

This consisted of two measures adopted and modified from Füchslin et al. (2019) and Kang et al. (2023), where responses ranged on a seven-point scale from 1 (never) to 7 (most of the time). *Technical Information Source* was self-reported with six items measuring information-seeking from scientists, government officials, news media, social media, business leaders, and political party leaders (heatwave *α* = 0.91, salmonella *α* = 0.90). *Personal Information Source* comprised of five items assessing information-seeking from leaders at places of worship, family, friends, colleagues, and acquaintances (heatwave *α* = 0.93, salmonella *α* = 0.93).

#### Social trust and distrust

Social Trust and distrust items were adopted from Gauchat (2011), with responses ranging from 1 (*strongly disagree*) to 7 (*strongly agree). Social Trust* self-reported respondents’ perceptions of whether people were “Helpful,” “Fair,” and “Trusted” (heatwave *α* = 0.82, salmonella *α* = 0.84). *Social Distrust* self-reported perceptions across three items: “People Usually Lookout for Themselves,” “People Will Often Take Advantage of Others,” and “One Can’t Be Too Careful” (heatwave *α* = 0.76, salmonella *α* = 0.80).

#### Perceptions of scientists’ motivation

One measure with two items self-reported by participants’ perceptions of scientists being influenced by a) best available evidence and b) public interest, with responses from 1 (strongly disagree) to 7 (strongly agree; heatwave *α* = 0.79, salmonella *α* = 0.72). This self-reported measure was adopted and modified from Hunt & Wald (2020).

#### Support for environmental action and vaccination

Participants in both conditions were asked to respond to the support questions. Previous work has established a strong association between perceived source credibility and science-informed policy support and evidence of significant positive spillover beliefs and behaviors across health and environmental contexts (Bancale et al., 2024). Thus, we hypothesized that if hedging increased perceived source credibility and positive beliefs about environmental health specialists, we might see an increase in support for both environmental action and vaccination support. Following recommendations by other scholars who have examined behavioral spillover effects, we designed these measures as distinct, unrelated components of environmental health behavior (Galizzi and Whitmarsh 2019; Nash et al. 2017). The support measures were created using modified measures from Füchslin et al. (2019) and Kang et al. (2023), with responses ranging from 1 (*strongly disagree*) to 7 (*strongly agree*). Support for *Environmental Action (EA)* consisted of six items (e.g., “Environmental measures are Important,” “Environmental measures is Necessary,” “Environmental Practices Benefit Outweigh Costs,” “Government Should Create Environmental Policies,” “Government Should Mandate Environmental Policies,” “Businesses Should Mandate Environmental Policies”; heatwave *α* = 0.95, salmonella *α* = 0.95). *Vaccination Support* included six items addressing necessity and mandate support for rabies, avian flu, and COVID-19 vaccines (heatwave *α* = 0.94, Salmonella *α* = 0.94).

#### Science beliefs/attitudes

Empirical clustering techniques for audience segmentation simplifies complex data, typically consists of psychosocial predictors such as beliefs and values, into meaningful interpretable answers (Smith, 2017). To that end, we also included measures of participants’ beliefs about science, drawn from previous research on the theory of deference to scientific authority (Brossard & Nisbet, 2007; Nisbet and Markowitz, 2014) and public attitudes toward science, including the works of Besley (2018), Gauchat (2011), Kang et al. (2023), Rabinovich and Morton (2012), and Sturgis and Allum (2004). The theory of deference to scientific authority suggests that optimistic and pessimistic beliefs about science develop early and remain stable throughout adulthood. If our findings support this theory, groups with high scores on perceived scientific source credibility should also be high on science optimism beliefs and groups skeptical of perceived scientific source credibility should report high scores on the science pessimism beliefs items. Participants rated statements from 1 (*strongly disagree*) to 7 (*strongly agree*). *Science Optimism* comprised six items including “Science Create Opportunities for the Next Generation,” “Technology Creates Opportunities for the Next Generation,” “Benefits of Scientific Research Have Outweighed the Harmful Results,” “Technological Progress Is Essential to U.S. Economic Competitiveness,” “Science and Technology Provide the Best and Most Reliable Knowledge About the World,” and “Role of Science is to Uncover Truth” (heatwave *α* = 0.87, salmonella *α* = 0.80). *Science Skepticism* was self-reported with four items: “Science Change Life Too Fast,” “Technology Changes Life Too Fast,” “We Give Too Much Value to Scientific and Technologic Knowledge,” and “Science and Technology Create an Artificial and Inhuman Lifestyle” (heatwave *α* = 0.81, salmonella *α* = 0.78).

### Latent class analysis

Both Latent Class Analysis (LCA) and Latent Profile Analysis (LPA) are person-centered approaches used to identify meaningful subgroups within data; however, each relies on underlying distributional assumptions that influence the quality of the resulting segments (Oberski et al., 2016). LCA is designed for categorical data but can accommodate continuous data, whereas LPA is restricted to continuous data only (Weller et al., 2020; Smith, 2017; Oberski et al., 2016). LCA offers several advantages over LPA, even when analyzing continuous data. Specifically, LCA does not impose distributional assumptions, whereas LPA requires responses to be normally distributed (Oberski et al., 2016). Additionally, LCA can identify extreme response patterns in highly skewed data—a capability unavailable with the linear models underlying LPA (Oberski et al., 2016). Because LCA estimates the distribution of latent variables directly from the data rather than assuming parametric forms, it is more robust for the discrete, bounded nature of Likert scale responses. LCA is considered the optimal technique for audience segmentation (Maibach et al., 2011). This approach ultimately yields more accurate and interpretable audience segments.

We used the PoLCA package in R to apply the LCA for the source credibility items (Linzer & Lewis, 2011). LCA was first introduced by Lazarsfeld (1950) and has undergone several phases of evolution that have impacted reporting practices. Therefore, to ensure consistency with the most up-to-date LCA practices, we follow the guidelines Weller et al. (2020) laid out in their review. It is essentially a person-centered mixture modeling approach that identifies latent subpopulations in a sample based on responses from the observed variables (Muthén & Muthén, 2000). It has been a popular approach amongst scholars in science and health communication to segment audiences (Besley, 2018; Maibach et al., 2009; Metag & Schafer, 2018; Richardson et al., 2023; Schäfer et al., 2018; Smith, 2017). We specified maximum likelihood estimation with robust standard errors (Muthén et al., 2015). We followed Weller et al.‘s (2020) and Sinha et al.’s (2021) best practices in reporting the fit and diagnostics criteria for model selection. We initially estimated a one-class model to identify scientists’ source credibility profiles for heatwaves and salmonella (Aim 1). Then, we added classes till a best-fit model was identified.

### Class identification

To address RQ 1 and 2, we sought to (a) identify the latent profile of U.S. adults based on their perceptions of scientific source credibility and (b) determine how these subgroups might emerge and vary across two environmental health contexts: heatwaves and zoonotic diseases. The latent class models were examined based on the fit of the theoretical understanding of source credibility (Horton et al., 2016, 2025; Hunt & Wald, 2020), which includes three components: competence, integrity and goodwill, and the following statistical criteria: (a) the Akaike information criterion (AIC), where the lower AIC indicates better fit (Weller et al., 2020), (b) the Bayesian information criterion (BIC), where lower BIC indicates a better model fit (Nylund et al., 2007), and (c) consistent Akaike information criterion (CAIC) (Dziak et al., 2019). We also examined the diagnostic criteria of the six models. Prior work has used a cut-off criterion of 0.8 for entropy (which indicates the separation between classes), along with the latent class posterior probability being 0.80 or higher for the smallest class size (Weller et al., 2020).

Based on the evaluation of the fit criteria (Table 3), the 5-class model had the lowest values (those after tapering off). After class 7, the model became unstable. All the classes exhibit satisfactory class separation, and the 5-class model meets the prior criteria (Table 4).

**Table 3:** Model Fit Criteria.

Heatwave	AIC	BIC	CIAC	LL	DF	Chisq
1 Class	43,512.63	44,183.99	44,345.99	−21,594.3	162	4.80E + 35
2 Class	37,001.34	38,348.2	38,673.2	−18,175.7	325	1.52E + 28
3 Class	34709	36,731.36	37,219.36	−16,866.5	488	7.03E + 26
4 Class	32,972.02	35,669.88	36,320.88	−15,835	651	3.18E + 27
5 Class	32,448.7	35,822.06	36,636.06	−15,410.3	814	6.39E + 29
6 Class	31,963.93	36,012.8	36,989.8	−15,005	977	5.07E + 26
7 Class	31,721.53	36,445.91	37,585.91	−14,720.8	1140	1.74E + 25

*Salmonella*	AIC	BIC	CIAC	LL	DF	Chisq
1 Class	38,924.32	39,593.93	39,755.93	−19,300.2	162	5.66E + 45
2 Class	33,574.18	34,917.53	35,242.53	−16,462.1	325	2.06E + 38
3 Class	31,400.49	33,417.59	33,905.59	−15,212.2	488	4.15E + 36
4 Class	30,175.91	32,866.76	33,517.76	−14,437	651	9.77E + 29
5 Class	29,747.84	33,112.42	33,926.42	−14,059.9	814	1.07E + 24
6 Class	29,430.2	33,468.53	34,445.53	−13,738.1	977	1.11E + 24
7 Class	29,319.92	34,031.99	35,171.99	−13,520	1140	3.54E + 27

**Table 4:** Model Class Separations.

Heatwave	Smallest Class Percent	Smallest Class *n*	Entropy
1 Class	100	466	NA
2 Class	47	220	0.9858
3 Class	16	74	0.992021
4 Class	15	69	0.985155
5 Class	8	35	0.982034
6 Class	10	45	0.989564
7 Class	8	39	0.99019

*Salmonella*	Smallest Class Percent	Smallest Class *n*	Entropy
1 Class	100	461	NA
2 Class	40	186	0.988203
3 Class	25	114	0.974456
4 Class	18	81	0.983508
5 Class	6	28	0.984757
6 Class	5	23	0.988417
7 Class	4	19	0.983458

### Generalized linear regression models (GLM)

To answer RQ3 and 4 we examined a) how confidence in individual scientists and scientific institutions, attention to sources, general social trust, political and sociodemographic factors, and beliefs about scientists’ motivation influenced perceptions of scientists’ source credibility across different subgroups and varied contexts? And b) how whether the profiles were associated with support for protective behaviors, such as mitigating exposure to extreme heat and getting vaccinated. we estimated regression models. We modeled each outcome using generalized linear models (GLMs) with a Gaussian family and identity link in R (using the glm function from the stats package) (R Core Team, 4.3.3). Dependent variables were (a) perceived source credibility (with subscales for competence, integrity, and goodwill) and (b) support for environmental action to mitigate extreme heat and vaccination to mitigate zoonotic disease). For (b) and (c) two models were estimated, where model 1 included demographics only and model 2 included all other measures as well. Pooled sensitivity analyses combining both behavioral outcomes (support for environmental action to mitigate extreme heat and vaccination to mitigate zoonotic disease) confirmed the robustness of these models: the extreme heat outcome model explained a substantial proportion of variance (R² = 0.596, F(16, 449) = 41.47, *p* < 0.001), as did the zoonotic disease outcome model (R² = 0.397, F(16, 444) = 18.27, *p* < 0.001), supporting the analytical defensibility of treating these behaviors as distinct outcomes reported by condition. All outcomes were measured on 7-point Likert or dichotomous scales but treated as continuous for analysis, consistent with evidence that parametric regression is robust to ordinal Likert data (Norman, 2010). Independent variables included confidence in scientists, in general institutions, information-seeking (technical and personal sources), demographics, and latent class membership (Estranged to Enthusiasts). GLMs with a Gaussian identity link were used because the outcomes are continuous and approximately normal; this approach is equivalent to multiple linear regression and is recommended for normally distributed behavioral measures (Balaskas, 2024).

Furthermore, we assessed homogeneity of variance using Levene’s test, which was significant (*p* < 0.05), indicating heteroskedasticity across groups. To account for this violation, we obtained heteroskedasticity-consistent (robust) standard errors in all models (Roy et al., 2025). Using robust standard errors (a “sandwich” estimator) ensures reliable inference even when the assumption of equal variances is not met (Roy et al., 2025). All tests were two-tailed with *α* = 0.05, and parameter estimates (regression coefficients) were reported for each predictor’s association with the outcome.

## Results

**Fig. 1:**
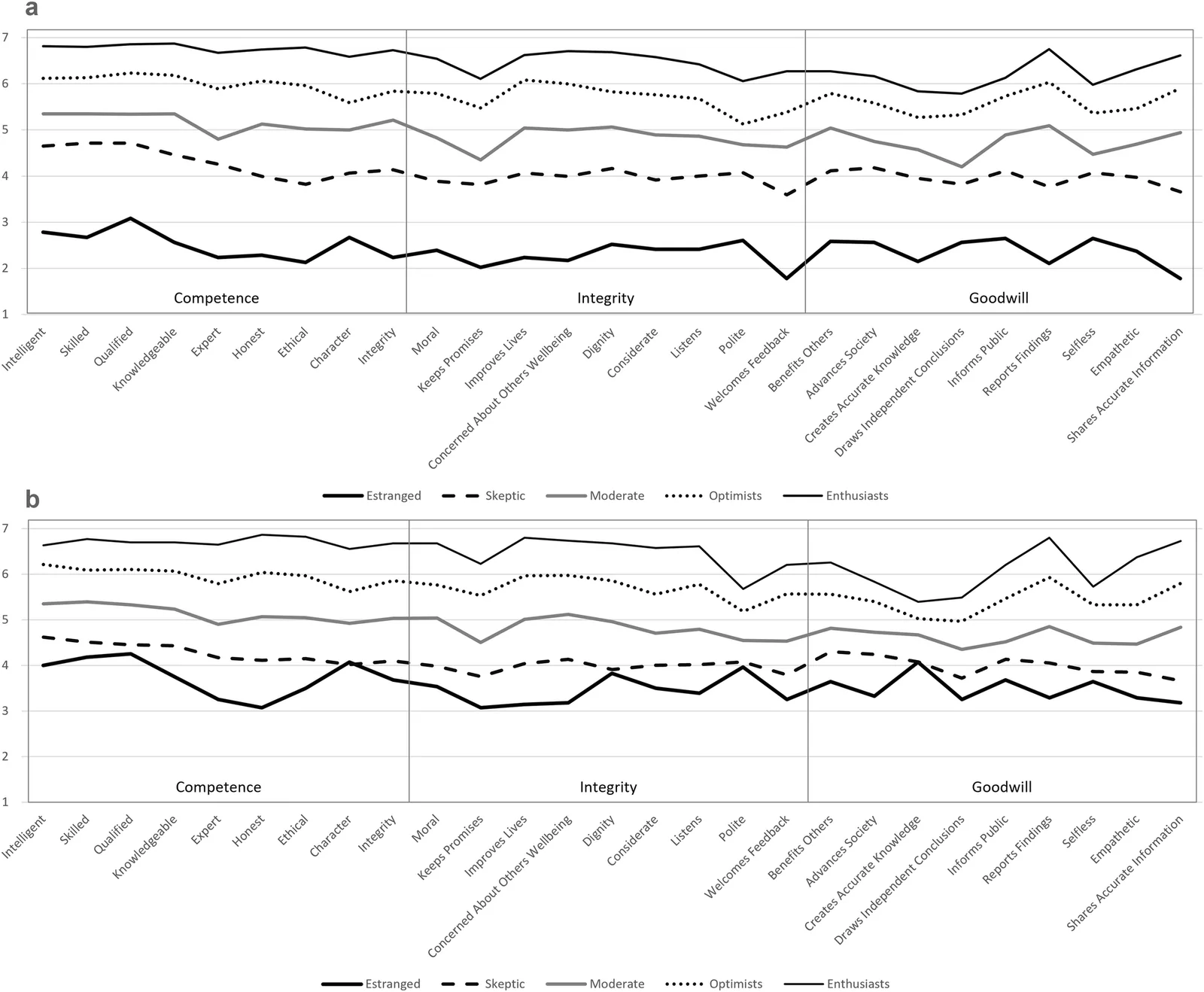
Class mean averages were calculated for the five classes across the individual items with the three dimensions of source credibility, namely competence, integrity, and goodwill. **a** shows class mean averages for source credibility perceptions for EH scientists working on heatwave. **b** shows class mean averages for source credibility perceptions for EH scientists working on Salmonella.

Using the PoLCA, we identified five latent classes for heatwave and *Salmonella*: ‘Estranged,’ ‘Skeptic,’ ‘Moderate,’ ‘Optimistic,’ and ‘Enthusiasts.’ Fig. 1a, b show the average class responses for the variables in the source credibility for heatwave and *salmonella*, respectively. Where Enthusiasts have the highest perceptions of scientists’ credibility and Estranged have the lowest. These findings address RQ1 and provide evidence to suggest that there are multiple subgroups among the US public that differ in important ways regarding perceived scientific source credibility. To address RQ2, we conducted a series of comparative analyses across the five audience segments (here on out called classes). In the following section, we provide a descriptive evaluation of differences among the classes, followed by results from a series of ANOVA tests estimating differences within the U.S. public based on their perceptions of environmental health (EH) specialists’ source credibility across two topical conditions: heatwave and *Salmonella*.

### Enthusiasts (Advocates, science elites, engaged)

Enthusiasts represent the segment most strongly engaged with science. They are distinguished by their exceptionally positive perceptions of scientists across key dimensions of credibility—competence, integrity, and goodwill—alongside highly optimistic attitudes toward science and minimal skepticism. This group demonstrated the strongest support for environmental action and health behaviors. Notably, Enthusiasts also reported elevated social trust and general confidence in institutions, extending to their perceptions of scientists’ motivations as being driven by the best available evidence and the public’s interest. Enthusiasts’ exceptionally positive perceptions of scientists and strong institutional confidence align with research linking high trust in scientific institutions to liberal political ideology (Besley, 2015; Cologna et al., 2025; Gauchat, 2011; Gauchat et al., 2017) and higher education levels (Hayat et al., 2019; Metzger & Flanigan, 2015). The demographic portrait reveals a clustering characterized by predominantly White/Caucasian identity, relatively higher income levels, and a tendency toward liberal ideology.

### Optimists (Science optimists, conditional supporter/advocates)

Optimists demonstrate moderately high engagement with science, with credibility perceptions—encompassing competence, integrity, and goodwill—approaching Enthusiasts. However, their attitudes exhibit notable context-dependency: optimists reported higher scientific optimism and stronger support for environmental action than Enthusiasts for salmonella, while displaying lower optimism and reduced vaccination support for heatwaves, though levels remained relatively high. This variable support reflects positive science attitudes sensitive to contextual factors and issue framing (Besley, 2015; Cologna et al., 2022), paralleling the benefit-focused conditional acceptance observed in Nisbet and Markowitz (2014), Conflicted segment. Demographically, optimists share a similar profile with Enthusiasts—predominantly White/Caucasian with mixed gender and age distributions—though leaning slightly more conservative politically.

### Moderates (Ambivalent, disengaged)

Moderates represent mid-range engagement with science, characterized by intermediate credibility perceptions and moderate scientific optimism. Their support demonstrated stark context-dependency: moderates mirrored Enthusiasts’ support for environmental action in the case of salmonella but showed significantly lower support for heatwave interventions. They reported intermediate institutional confidence and balanced reliance on technical and personal information sources, reflecting susceptibility to biased assimilation depending on issue salience (Lord et al., 1979). Occupying a middle ground in polarized contexts (Hamilton et al., 2015), moderates exhibited moderate political ideology—positioned between Enthusiasts and skeptics/estranged—with mixed sociodemographic characteristics and no strong demographic clustering. This persuadable centrist profile parallels characteristics of Nisbet and Markowitz’s (2014) Conflicted segment, though moderates appear less consistently ambivalent and more responsive to specific issue framing.

### Skeptics (Science skeptics)

Skeptics represent notably low engagement with science, distinguished by diminished credibility perceptions across competence, integrity, and goodwill, alongside significantly lower scientific optimism and elevated skepticism, particularly pronounced in the Salmonella condition. They demonstrated reduced support for environmental action and vaccination across both contexts, coupled with lower confidence in scientists’ motivations and diminished institutional trust. Skeptics’ conservative ideology and questioning of scientific competence align with established patterns linking conservatism to scientific skepticism (Gauchat, 2012; Gauchat et al., 2017) and distrust of scientists’ competence, integrity, and goodwill (Hunt & Wald, 2018, 2020). Their reduced reliance on technical sources and lower confidence suggest inferences about scientists’ political affiliations shape credibility judgments (Kotcher et al., 2017; Vraga et al., 2018), making them more likely to reject scientific recommendations (Nisbet and Markowitz, 2014; Pechar et al., 2018). Skeptics often did not differ significantly from the estranged on key measures and shared characteristics with Nisbet and Markowitz’s (2014) Scientific Pessimists, though with stronger partisan political dimensions. Demographically, they cluster around conservative ideology, higher Republican affiliation, greater Black/African American representation (particularly in heatwave), Midwest residence (particularly in salmonella), lower educational attainment, and middle-aged and older (~50 + ).

### Estranged (Deniers, alienated, disengaged)

Estranged represent the most disconnected segment from science, distinguished by the lowest credibility perceptions across competence, integrity, and goodwill, and the lowest scientific optimism, particularly pronounced in the heatwave condition. They demonstrated dramatically lower support for environmental action across both contexts—the largest negative effects among all classes—with minimal confidence in scientists’ motivations and the lowest institutional trust broadly. As the least likely to seek technical information sources, the estranged embody the strongest conservative skepticism of scientific competence (Gauchat, 2012) and exhibit polarized responses to environmental issues (Funk et al., 2019a; Hamilton et al., 2015; Nisbet et al., 2015), with distrust extending across scientific and general institutions (Gauchat et al., 2017). This profile parallels Nisbet and Markowitz’s (2014) Disengaged segment but reflects deeper ideological resistance rather than mere lack of information. Demographically, they cluster around the most conservative ideology, strongest Republican affiliation (particularly in salmonella), lower educational attainment, middle-aged and older (~50 + ), and higher male representation, with higher income negatively associated with class membership.

Significant differences across the five latent classes were observed for most psychosocial, cognitive, and behavioral factors in both the Heatwave and *Salmonella* topics. Levene’s test indicated significant heterogeneity of variance. As a result, we employed the Kruskal-Wallis test with post hoc Dunn tests to ensure appropriate non-parametric comparisons (Dinno, 2015; Van Hecke, 2010). Results are reported in Table 5 a, b.

**Table 5:** a: Mean class differences for source credibility perceptions context of heatwave; b: Mean class differences for source credibility perceptions context of Salmonella.

		Estranged (*n* = 35)		Skeptics (*n* = 125)		Moderate (*n* = 125)		Optimists (*n* = 95)		Enthusiasts (*n* = 86)	
**Heatwave**	*p*−value	*M*	SD	*M*	SD	*M*	SD	*M*	SD	*M*	SD
Competence i	<0.001	2.67^a^	1.12	4.59^b^	0.79	5.44^c^	0.55	6.26 ^d^	0.48	6.81^e^	0.28
Openness i	<0.001	2.23^a^	0.73	4.07^b^	0.42	5.13^c^	0.35	5.80 ^d^	0.41	6.64^e^	0.37
Benevolence i	<0.001	2.31^a^	0.87	4.06^b^	0.58	4.94^c^	0.44	5.70 ^d^	0.43	6.26^e^	0.71
Science Optimism	<0.001	3.49^a^	1.37	4.63^b^	0.72	5.20^c^	0.70	5.82 ^d^	0.66	6.35^e^	0.82
Science Skepticism	0.001	4.76^a^	1.74	4.56^b^	0.88	4.27^b,c^	1.08	4.09^c^	1.36	4.24^b,c^	1.87
Environmental Health Support i	<0.001	2.23^a^	1.36	4.08^b^	1.20	5.08^c^	1.03	5.80 ^d^	0.88	6.21 ^d^	1.20
Vaccination Support ii	<0.001	2.38^a^	1.50	3.87^b^	1.29	4.58^c^	1.20	5.10 ^d^	1.17	5.85 ^d^	1.27
Technical Information Source i	<0.001	2.04^a^	1.33	2.62^b^	1.28	2.93^c^	1.22	3.48^c^	1.47	4.05 ^d^	1.66
Personal Information Source i	0.034	2.49^a^	1.54	2.58^a,b^	1.25	2.54^a,b^	1.32	2.86^b,c^	1.62	3.27^c^	1.88
Social Trust i	<0.001	3.50^a^	1.44	4.25^b^	1.01	4.65^c^	1.09	4.84 ^d^	1.15	5.56^e^	1.13
Social Distrust i	0.208	4.87^a^	1.56	4.77^a^	1.04	4.75^a^	1.15	4.79^a^	1.26	4.68^a^	1.65
Scientists Influenced i	<0.001	2.57^a^	1.25	4.30^b^	0.76	5.39^c^	0.67	6.13 ^d^	0.65	6.73^e^	0.50
Confidence in General Institutions i	<0.001	2.46^a^	1.25	3.24^b^	1.11	3.65^c^	1.15	4.06 ^d^	1.28	4.67 ^d^	1.57
Confidence in Scientists i	<0.001	1.95^a^	1.21	4.08^b^	1.19	5.16^c^	0.98	5.07 ^d^	1.12	6.60^e^	0.85
Gender ii	0.990	1.53^a^	0.50	1.54^a^	0.50	1.44^a^	0.50	1.53^a^	0.50	1.47^a^	0.50
Age ii	0.898	50.98^a^	18.01	53.59^a^	19.34	56.16^a^	17.76	55.25^a^	17.45	53.75^a^	15.76
Hispanic ii	0.984	0.12^a^	0.32	0.09^a^	0.29	0.08^a^	0.27	0.09^a^	0.29	0.10^a^	0.30
Race (White/Caucasian) ii	0.774	0.74^a^	0.44	0.75^a^	0.43	0.83^a^	0.38	0.78^a^	0.42	0.76^a^	0.43
Race (Black/African America) ii	0.506	0.16^a^	0.37	0.17^a^	0.38	0.07^a^	0.26	0.16^a^	0.36	0.17^a^	0.38
Race (Asian, Native Americans, etc.) ii	0.461	0.09^a^	0.29	0.08^a^	0.27	0.10^a^	0.30	0.07^a^	0.25	0.07^a^	0.26
Political Ideology ii	<0.001	5.49^a^	1.84	4.95^b^	1.58	4.26^c^	1.71	3.76^c,d^	1.85	3.28 ^d^	1.86
Political Party i	<0.001	2.07^a^	0.55	2.08^a^	0.74	1.90^b^	0.80	1.69^c^	0.81	1.46^c^	0.77
Education ii	0.767	3.56^a^	1.61	3.48^a^	1.37	3.73^a^	1.40	3.37^a^	1.46	4.04^a^	1.49
Income ii	0.947	2.58^a^	1.58	2.62^a^	1.40	2.67^a^	1.41	2.72^a^	1.50	2.63^a^	1.44
Region (South) ii	0.113	0.51^a^	0.51	0.38^a^	0.49	0.27^b^	0.45	0.39^c^	0.49	0.38^c^	0.49
Region (West) ii	0.162	0.28^a^	0.45	0.20^a^	0.40	0.23^a^	0.42	0.19^a,b^	0.39	0.14^b^	0.35
Region (Midwest) ii	0.079	0.12^a^	0.32	0.19^c^	0.40	0.31^b^	0.47	0.22^c^	0.42	0.31^b^	0.46
Region (Northeast) ii	0.195	0.09^a^	0.29	0.22^c^	0.42	0.18^b^	0.39	0.20^c^	0.41	0.18^c^	0.39

		Estranged (*n* = 28)		Skeptics (*n* = 53)		Moderate (*n* = 103)		Optimists (*n* = 181)		Enthusiasts (*n* = 96)	
Salmonella	*p*-value	*M*	SD	*M*	SD	*M*	SD	*M*	SD	M	SD
Competence i	<0.001	4.17^a^	0.83	5.28^a^	0.52	5.76^b^	0.36	6.59^c^	0.67	6.43 ^d^	0.44
Openness i	<0.001	3.73^a^	0.65	4.90^a^	0.33	5.77^b^	0.24	6.58^c^	0.43	5.78 ^d^	0.47
Benevolence i	<0.001	3.72^a^	0.75	4.63^a^	0.46	5.46^b^	0.42	6.15^c^	0.58	5.47 ^d^	0.50
Science Optimism i	<0.001	4.36^a,b^	0.84	4.91^a^	0.67	5.51^b^	0.55	6.21^c^	0.60	5.80 ^d^	0.59
Science Skepticism i	0.001	4.52^a^	1.02	4.48^a^	0.88	4.11^a^	1.28	4.12^b^	1.64	3.97^b^	1.22
Environmental Health Support ii	<0.001	3.29^a,b^	1.46	4.13^a^	1.37	4.96^b^	1.41	5.88^c^	1.27	5.11 ^d^	1.47
Vaccination Support i	<0.001	3.23^a^	1.33	3.85^a^	1.32	4.36^a^	1.43	5.25^b^	1.55	4.12^c^	1.51
Technical Information Source i	0.001	2.71^a,b^	1.44	2.76^c^	1.31	2.78^a,c^	1.21	3.75^a^	1.87	3.13^a^	1.45
Personal Information Source i	0.022	2.80^a^	1.57	2.55^b^	1.31	2.49^b,c^	1.38	3.31^b,c^	2.01	2.69^a,c^	1.52
Social Trust i	<0.001	3.93^a,b^	1.10	4.44^a^	0.91	4.75^b^	1.22	5.22^c^	1.35	5.01^c^	1.24
Social Distrust i	0.074	4.71^a^	1.24	4.77^a^	1.00	4.60^a^	1.28	5.12^b^	1.29	4.47^a^	1.43
Scientists Influenced i	<0.001	3.84^a^	1.05	5.00^a^	0.65	5.76^b^	0.57	6.62^c^	0.74	6.30 ^d^	0.66
Confidence in General Institutions i	0.003	3.31^a^	1.22	3.50^a^	1.23	3.65^a,b^	1.22	4.24^a,b^	1.59	3.81^c^	1.19
Confidence in Scientists i	<0.001	3.75^a^	1.44	4.64^a^	0.87	5.56^a^	0.87	6.42^b^	0.89	5.80^c^	0.96
Gender ii	0.479	1.43^a^	0.50	1.54^a^	0.50	1.57^a^	0.50	1.48^a^	0.50	1.51^a^	0.50
Age ii	0.049	50.84^a^	18.03	55.35^a,b^	18.17	58.55^b,c^	18.06	53.52^c^	17.79	58.62^b,c^	15.61
Hispanic ii	0.383	0.08^a^	0.27	0.08^a^	0.28	0.00^a^	0.00	0.10^a^	0.30	0.08^a^	0.27
Race (White/Caucasian) ii	0.652	0.75^a^	0.43	0.76^a^	0.43	0.85^a^	0.36	0.81^a^	0.40	0.81^a^	0.39
Race (Black/African America) ii	0.702	0.14^a^	0.35	0.13^a^	0.34	0.08^a^	0.27	0.12^a^	0.33	0.12^a^	0.33
Race (Asian, Native Americans, etc.) ii	0.779	0.10^a^	0.31	0.11^a^	0.32	0.07^a^	0.25	0.07^a^	0.26	0.07^a^	0.25
Political Ideology i	<0.001	4.95^a^	1.84	4.73^a^	1.66	4.30^a^	1.79	3.55^b^	2.13	4.08^c^	1.89
Political Party ii	0.002	2.05^a,b^	0.69	1.93^a^	0.74	1.83^a,b^	0.77	1.56^b,c^	0.78	1.76^c^	0.82
Education i	0.001	3.32^a^	1.37	3.50^b^	1.29	3.57^b^	1.51	3.80^b^	1.57	3.91^b^	1.64
Income ii	0.386	2.88^a^	1.40	2.60^a^	1.48	2.93^a^	1.69	2.54^a^	1.59	2.80^a^	1.41
Region (South) ii	0.650	0.34^a^	0.48	0.45^a^	0.50	0.39^a^	0.49	0.27^a^	0.45	0.43^a^	0.50
Region (West) ii	0.531	0.23^a^	0.43	0.21^a^	0.41	0.20^a^	0.40	0.26^a^	0.44	0.21^a^	0.41
Region (MidWest) ii	0.315	0.29^a^	0.45	0.24^a^	0.43	0.21^a^	0.41	0.31^a^	0.47	0.21^a^	0.41
Region (North East) ii	0.220	0.14^a^	0.35	0.10^a^	0.31	0.21^a^	0.41	0.15^a^	0.36	0.15^a^	0.36

Kruskal-Wallis with Dunn post-hoc (i) were used where Levene’s test indicated unequal variances. ANOVA with Tukey HSD (ii) was applied when variance homogeneity held. *P*-value indicates a significant in-group difference based on classes.

*M* Mean, *SD* Standard Deviation.

^a,b,c,d,e^ Means that do not share a superscript letter are significantly different from each other.

For the Heatwave topic, Kruskal-Wallis tests indicated that Competence (χ²(4) = 341.31, *p* < 0.001), Integrity (χ²(4) = 402.66, *p* < 0.001), Goodwill (χ²(4) = 366.72, *p* < 0.001), Science Optimism (χ²(4) = 235.50, *p* < 0.001), Science Skepticism (χ²(4) = 18.53, *p* = 0.001), EH support (χ²(4) = 230.01, *p* < 0.001), Vaccination Support (χ²(4) = 153.32, *p* < 0.001), Technical Information Sources (χ²(4) = 71.91, *p* < 0.001), Personal Information Sources (χ²(4) = 10.44, *p* = 0.034), Social Trust (χ²(4) = 84.81, *p* < 0.001), Scientific Influence (χ²(4) = 331.88, *p* < 0.001), Confidence in General Institutions (χ²(4) = 94.99, *p* < 0.001), and Confidence in Scientific Institutions (χ²(4) = 267.27, *p* < 0.001) differed significantly across classes, whereas Social Distrust and most demographic variables did not.

Post-hoc Dunn test revealed a clear gradient and class separation from Estranged to Enthusiasts, with Estranged consistently scoring lower perceptions of scientists across credibility dimensions and Enthusiasts higher, indicating that all five classes were significantly different on these measures. Intermediate classes (Skeptics, Moderate, Optimists) exhibited progressive increases in scores, with some overlapping for certain factors such as seeking Personal Information Sources, reflecting partial similarity between classes. Overall, the factors showing the strongest differentiation among Heatwave classes were Integrity, Goodwill, Competence, Science Optimism, attitude towards EH behaviors (EH support), and Scientist’s Motivation.

For the *Salmonella* condition, significant class differences were observed for Competence (χ²(4) = 296.82, *p* < 0.001), Integrity (χ²(4) = 376.56, *p* < 0.001), Goodwill (χ²(4) = 301.04, *p* < 0.001), Science Optimism (χ²(4) = 207.24, *p* < 0.001), Science Skepticism (χ²(4) = 17.94, *p* = 0.001), EH Support (χ²(4) = 133.62, *p* < 0.001), Vaccination Support (χ²(4) = 52.44, *p* < 0.001), Technical Information Sources (χ²(4) = 19.69, *p* = 0.001), Personal Information Sources (χ²(4) = 11.50, *p* = 0.022), Social Trust (χ²(4) = 62.08, *p* < 0.001), Scientists Influence (χ²(4) = 284.94, *p* < 0.001), Confidence in General Institutions (χ²(4) = 15.78, *p* = 0.003), and Confidence in Scientists (χ²(4) = 210.81, *p* < 0.001). Like Heatwave, post-hoc Dunn test indicated that the classes with lower credibility perception (Estranged and Skeptics) were not always significantly different from each other, but were consistently lower than Moderate, Optimists, and Enthusiasts. Factors such as Integrity, Goodwill, Competence, Science Optimism, EH Support, Scientists Motivation, and Confidence in Scientists exhibited the largest separation between classes. Other variables, including Social Distrust, demographics, and some political indicators, showed smaller or inconsistent differences across classes.

To answer RQ3 (see Table 6), for the heatwave condition, GLM regression revealed that perceived scientists’ motivation was the strongest factor associated with all three dimensions of source credibility (*p* < 0.001), with the largest effect observed for perception of scientists’ competence (*b* = 0.65, *p* < 0.001). Institutional confidence in scientists was significantly associated with all three credibility dimensions (competence: *b* = 0.17, *p* < 0.001; integrity: *b* = 0.23, *p* < 0.001; goodwill: *b* = 0.25, *p* < 0.001). Social trust was positively associated with perception of scientists’ integrity (*b* = 0.06, *p* < 0.05) and goodwill (*b* = 0.07, *p* < 0.05), while social distrust was only associated with perception of scientists’ integrity (*b* = 0.06, *p* < 0.01). Technical information source seeking was significantly associated with perception of scientists’ goodwill (*b* = 0.08, *p* < 0.01), and general institutional confidence was positively associated with both perception of scientists’ integrity (*b* = 0.07, *p* < 0.05) and goodwill (*b* = 0.08, *p* < 0.01). Among demographic variables, higher education was negatively associated goodwill (*b* = −0.05, *p* < 0.05), and Hispanic ethnicity was negatively associated with integrity (*b* = −0.21, *p* < 0.05). The models explained substantial variance across all three credibility dimensions (competence: *R²* = 0.70; integrity: *R²* = 0.81; goodwill: *R²* = 0.78).

**Table 6:** GLM regression for DV perception of source credibility (competence, integrity, goodwill).

		Heatwave								Salmonella								
	Competence			Integrity			Goodwill			Competence			Integrity			Goodwill		
*Predictors*	*Estimates*	*std. Error*	*Statistic*	*Estimates*	*std. Error*	*Statistic*	*Estimates*	*std. Error*	*Statistic*	*Estimates*	*std. Error*	*Statistic*	*Estimates*	*std. Error*	*Statistic*	*Estimates*	*std. Error*	*Statistic*
(Intercept)	1.26 ^**^	0.39	3.27	0.37	0.3	1.25	0.74 ^*^	0.31	2.36	1.37 ^***^	0.35	3.96	0.39	0.29	1.33	0.77 ^*^	0.31	2.48
Gender (Woman = 2)	−0.01	0.07	−0.1	0.02	0.06	0.39	0.05	0.06	0.8	0.12	0.06	1.85	0.07	0.05	1.4	0.12 ^*^	0.06	2.15
Political Party (Republican = 3)	0	0.05	0.03	−0.04	0.04	−1.06	−0.05	0.04	−1.21	−0.04	0.04	−0.91	−0.05	0.04	−1.53	−0.04	0.04	−0.97
Political Ideology (Extremely Conservative = 7)	0	0.02	−0.13	−0.01	0.02	−0.58	−0.02	0.02	−1.24	0	0.02	−0.07	0.01	0.02	0.71	0	0.02	0.3
Education (Graduate/Professional = 7)	−0.02	0.02	−0.86	−0.03	0.02	−1.81	−0.05 ^*^	0.02	−2.35	0.06 ^**^	0.02	2.87	0	0.02	0	0.01	0.02	0.36
Income (> $150,000 = 7)	0.03	0.02	1.31	−0.01	0.02	−0.53	−0.01	0.02	−0.49	0.01	0.02	0.33	0.01	0.02	0.69	0	0.02	0.11
Region (South) (ref: North)	−0.02	0.1	−0.16	−0.06	0.08	−0.84	0.03	0.08	0.4	−0.06	0.09	−0.61	−0.16 ^*^	0.08	−2.15	−0.12	0.08	−1.49
Region (Midwest)	0.06	0.11	0.57	−0.05	0.08	−0.64	−0.03	0.09	−0.3	−0.06	0.1	−0.66	−0.18 ^*^	0.08	−2.2	−0.04	0.09	−0.46
Region (West)	−0.05	0.11	−0.47	−0.1	0.09	−−1.08	−0.1	0.09	−1.08	−0.12	0.1	−1.21	−0.14	0.08	−1.64	−0.06	0.09	−0.69
Race (Black/African Americans) (ref: White/Caucasian)	−0.04	0.11	−0.41	0.1	0.08	1.23	0.07	0.09	0.81	−0.06	0.1	−0.59	−0.14	0.09	−1.65	−0.01	0.09	−0.09
Race (Asian, Native Americans, etc.)	−0.22	0.14	−1.6	−0.07	0.11	−0.68	−0.07	0.11	−0.66	0.1	0.12	0.86	0.16	0.1	1.65	0.12	0.1	1.19
Hispanic (Yes = 2)	−0.14	0.13	−1.07	−0.21 ^*^	0.1	−2.09	−0.03	0.11	−0.24	0.19	0.13	1.45	−0.06	0.11	−0.56	−0.1	0.12	−0.87
Age	0	0	−1.72	0	0	−0.14	0	0	−0.59	0	0	0.84	0	0	0.78	0	0	−1.28
Scientists Motivations	0.65 ^***^	0.04	18.4	0.53 ^***^	0.03	19.49	0.42 ^***^	0.03	14.85	0.50 ^***^	0.03	15.84	0.48 ^***^	0.03	17.87	0.39 ^***^	0.03	13.67
Personal Information Source	0	0.04	0.06	−0.01	0.03	−0.25	−0.01	0.03	−0.4	0	0.03	0.01	0.01	0.02	0.5	−0.01	0.03	−0.21
Technical Information Source	−0.01	0.04	−0.17	0.05	0.03	1.74	0.08 ^**^	0.03	2.87	0.03	0.03	0.91	0.02	0.03	0.67	0	0.03	−0.03
Social Trust	0.02	0.04	0.58	0.06 ^*^	0.03	2.17	0.07 ^*^	0.03	2.35	0.07 ^*^	0.03	2.18	0.09 ^**^	0.03	3.25	0.08 ^**^	0.03	2.66
Social Distrust	0.05	0.03	1.51	0.06 ^**^	0.02	2.58	0.04	0.03	1.62	0.05	0.03	1.8	0.10 ^***^	0.02	4.31	0.08 ^**^	0.03	3.18
Confidence in General Institutitons	−0.04	0.04	−1.21	0.07 ^*^	0.03	2.52	0.08 ^**^	0.03	2.7	−0.11 ^***^	0.03	−3.58	0.02	0.03	0.85	0.05	0.03	1.89
Confidence in Scientists	0.17 ^***^	0.03	5.28	0.23 ^***^	0.03	9.18	0.25 ^***^	0.03	9.25	0.17 ^***^	0.03	5.6	0.24 ^***^	0.02	9.53	0.24 ^***^	0.03	9.13
Observations	466			466			466			461			461			461		
R^2^	0.701			0.814			0.778			0.628			0.739			0.666		

* *p* < 0.05 ** *p* < 0.01 *** *p* < 0.001.

For *Salmonella* (see Table 6), perceived scientists’ influence again emerged as the strongest association across all source credibility dimensions (*p* < .001), with effects ranging from *b* = 0.39 to *b* = 0.50. Institutional Confidence in Scientists consistently predicted all three dimensions (competence: *b* = 0.17, *p* < 0.001; integrity: *b* = 0.24, *p* < 0.001; goodwill: *b* = 0.24, *p* < 0.001). Social trust was significantly associated with perception of scientists’ integrity (*b* = 0.09, *p* < 0.01) and goodwill (*b* = 0.08, *p* < 0.01), while social distrust was associated with perception of scientists’ integrity (*b* = 0.10, *p* < .001) and goodwill (*b* = 0.08, *p* < 0.01). Institutional confidence negatively associated with competence (*b* = −0.11, *p* < .001). Notably, education positively predicted with perception of scientists’ competence (*b* = 0.06, *p* < 0.01), gender associated with perception of scientists’ goodwill (*b* = 0.12, *p* < 0.05), and regional effects emerged for integrity perceptions (South: *b* = −0.16, *p* < 0.05; Midwest: *b* = −0.18, *p* < 0.05). The models accounted for considerable variance across all three source credibility dimensions for scientists (competence: *R²* = 0.63; integrity: *R²* = 0.74; goodwill: *R²* = 0.67).

Table 7a shows the GLM results for factors associated with support for environmental action in the heatwave category. Model 2 demonstrated that latent class membership was strongly associated with positive attitudes about environmental action and health behavior (EH). All classes showed significantly lower support compared to Enthusiasts: estranged (*b* = −3.61, *p* < 0.001), skeptics (*b* = −1.86, *p* < 0.001), moderates (*b* = −0.93, *p* < 0.001), and optimists (*b* = -0.31, *p* < 0.05). Conservative political ideology negatively predicted EH support (*b* = −0.12, *p* < 0.001), as did higher income (*b* = −0.11, *p* < 0.001). Women demonstrated greater support for EH behaviors than men (*b* = 0.26, *p* < 0.05). The model explained considerable variance (*R²* = 0.58).

**Table 7:** a: GLM regression for DVs science attitudes towards EH behaviors Heatwave; b: GLM regression for DVs science beliefs and attitudes towards EH behaviors Salmonella.

Heatwave	EA Model 1			EA Model 2			Vaccination Model 1			Vaccination Model 2		
*Predictors*	*Estimates*	*std. Error*	*Statistic*	*Estimates*	*std. Error*	*Statistic*	*Estimates*	*std. Error*	*Statistic*	*Estimates*	*std. Error*	*Statistic*
(Intercept)	6.88 ^***^	0.5	13.78	6.75 ^***^	0.38	17.78	6.55 ^***^	0.51	12.81	6.70 ^***^	0.44	15.17
Gender (Woman = 2)	0.1	0.14	0.69	0.26 ^*^	0.1	2.51	−0.38 ^**^	0.14	−2.66	−0.24 ^*^	0.12	−1.99
Political Party (Republican = 3)	−0.28 ^**^	0.09	−3.14	−0.1	0.07	−1.52	−0.25 ^**^	0.09	−2.71	−0.09	0.08	−1.12
Political Ideology (Extremely Conservative = 7)	−0.30 ^***^	0.04	−7.86	−0.12 ^***^	0.03	−4.01	−0.23 ^***^	0.04	−5.86	−0.08 ^*^	0.03	−2.27
Education (Graduate/Professional = 7)	−0.01	0.05	−0.18	−0.01	0.03	−0.32	0	0.05	0.07	−0.01	0.04	−0.26
Income (> $150,000 = 7)	−0.10 ^*^	0.05	−2.1	−0.11 ^***^	0.03	−3.35	−0.13 ^**^	0.05	−2.64	−0.14 ^***^	0.04	−3.44
Region (South) (ref: North)	−0.2	0.19	−1.08	−0.07	0.14	−0.54	−0.23	0.19	−1.2	−0.13	0.16	−0.82
Region (Midwest)	0.22	0.21	1.06	0.19	0.15	1.26	−0.18	0.21	−0.83	−0.21	0.18	−1.15
Region (West)	−0.4	0.21	−1.88	−0.18	0.16	−1.12	−0.57 ^**^	0.22	−2.63	−0.38 ^*^	0.18	−2.07
Race (Black/African Americans) (ref: White/Caucasian)	0.1	0.21	0.48	0.19	0.15	1.25	0.13	0.21	0.6	0.19	0.18	1.05
Race (Asian, Native Americans, etc.)	−0.2	0.26	−0.77	−0.05	0.19	−0.26	0.21	0.27	0.78	0.34	0.22	1.52
Hispanic (Yes = 2)	−0.03	0.25	−0.14	0.07	0.18	0.4	0.42	0.26	1.64	0.51 ^*^	0.21	2.38
Age	0	0	0.38	0	0	−0.45	0.01 ^*^	0	1.96	0.01	0	1.77
Classes (ref: Believers)												
Estranged				−3.60 ^***^	0.22	−16.67				−3.20 ^***^	0.25	−12.73
Skeptics				−1.85 ^***^	0.17	−11.15				−1.78 ^***^	0.19	−9.23
Moderates				−0.92 ^***^	0.17	−5.47				−1.14 ^***^	0.2	−5.83
Optimists				−0.31	0.16	−1.92				−0.67 ^***^	0.19	−3.63
Observations	466			466			466			466		
R^2^	0.221			0.58			0.16			0.415		

*Salmonella*	EA Model 1			EA Model 2			Vaccination Model 1			Vaccination Model 2		
*Predictors*	*Estimates*	*std. Error*	*Statistic*	*Estimates*	*std. Error*	*Statistic*	*Estimates*	*std. Error*	*Statistic*	*Estimates*	*std. Error*	*Statistic*
(Intercept)	6.67 ^***^	0.48	13.96	6.72 ^***^	0.45	14.97	7.06 ^***^	0.5	14.02	6.63 ^***^	0.5	13.34
Gender (Woman = 2)	0.19	0.13	1.45	0.16	0.12	1.35	−0.39 ^**^	0.14	−2.75	−0.41 ^**^	0.13	−3.09
Political Party (Republican = 3)	−0.32 ^***^	0.09	−3.61	−0.21 ^**^	0.08	−2.6	−0.38 ^***^	0.09	−3.99	−0.28 ^**^	0.09	−3.11
Political Ideology (Extremely Conservative = 7)	−0.38 ^***^	0.04	−10.39	−0.31 ^***^	0.03	−9.34	−0.18 ^***^	0.04	−4.73	−0.13 ^***^	0.04	−3.45
Education (Graduate/Professional = 7)	0.08	0.04	1.85	0.05	0.04	1.21	−0.01	0.05	−0.25	−0.03	0.04	−0.69
Income (> $150,000 = 7)	0	0.04	−0.04	0.01	0.04	0.37	0.01	0.05	0.25	0.03	0.04	0.73
Region (South) (ref: North)	−0.05	0.2	−0.26	0.01	0.18	0.04	−0.05	0.21	−0.24	0.01	0.19	0.04
Region (Midwest)	−0.03	0.21	−0.13	0.05	0.19	0.27	−0.21	0.23	−0.93	−0.19	0.21	−0.87
Region (West)	−0.01	0.22	−0.06	0.09	0.19	0.45	−0.32	0.23	−1.4	−0.26	0.21	−1.21
Race (Black/African Americans) (ref: White/Caucasian)	0.13	0.22	0.59	0.24	0.19	1.22	0.25	0.23	1.1	0.35	0.21	1.64
Race (Asian, Native Americans, etc.)	−0.08	0.25	−0.3	−0.01	0.22	−0.03	−0.05	0.26	−0.18	0	0.25	0.01
Hispanic (Yes = 2)	0.17	0.28	0.59	0.1	0.25	0.39	−0.07	0.29	−0.23	−0.1	0.28	−0.36
Age	−0.01	0	−1.43	−0.01 ^*^	0	−2.39	−0.01 ^**^	0	−2.91	−0.01 ^***^	0	−3.34
Classes (ref: Believers)												
Estranged				−1.53 ^***^	0.19	−8.08				−0.85 ^***^	0.21	−4.06
Skeptics				−0.76 ^***^	0.17	−4.46				−0.18	0.19	−0.96
Moderates				−0.05	0.18	−0.25				0.3	0.2	1.51
Optimists				0.52 ^**^	0.18	2.84				0.96 ^***^	0.2	4.77
Observations	461			461			461			461		
R^2^	0.301			0.457			0.151			0.27		

* *p* < 0.05 ** *p* < 0.01 *** *p* < 0.001.

Latent class membership strongly predicted vaccination support in to heatwave topic as well, accounting for behavior spillover. Compared to Enthusiasts (reference group), all classes showed significantly lower vaccination support: estranged (*b* = −3.20, *p* < 0.001), skeptics (*b* = −1.78, *p* < 0.001), moderates (*b* = −1.14, *p* < 0.001), and optimists (*b* = −0.67, *p* < 0.001). Among demographic predictors, conservative political ideology negatively predicted vaccination support (*b* = -0.08, *p* < 0.05), as did higher income (*b* = −0.14, *p* < .001). Women reported lower vaccination support than men (*b* = −0.24, *p* < 0.05), and participants from the Western region showed significantly lower support compared to the Northeast (*b* = −0.38, *p* < 0.05). Hispanic participants demonstrated higher vaccination support (*b* = 0.51, *p* < 0.05). The model explained substantial variance (*R²* = 0.42).

Table 7b shows the GLM results for factors associated with scientific beliefs and attitudes towards EH behaviors in the *Salmonella* category. Model 2 demonstrated that latent class membership strongly predicted environmental action and health behaviors (EH) support for those in the *Salmonella* category. The estranged class showed markedly lower support (*b* = −1.53, *p* < 0.001), followed by skeptics (*b* = −0.76, *p* < 0.001), while optimists reported significantly higher support than Enthusiasts (*b* = 0.52, *p* < 0.01). Moderates did not differ significantly from Enthusiasts. Conservative political ideology strongly negatively predicted EH support (*b* = −0.31, *p* < .001), as did Republican party affiliation (*b* = −0.21, *p* < 0.01). Older age was negatively associated with EH support (*b* = −0.01, *p* < 0.05). The model explained considerable variance (*R²* = 0.46).

In Model 2, latent class membership significantly predicted vaccination support for salmonella-related risks, though with different patterns than the heatwave condition. The estranged class showed the largest negative effect (*b* = −0.85, *p* < 0.001), while optimists reported significantly higher support than Enthusiasts (*b* = 0.96, *p* < 0.001). Conservative political ideology (*b* = −0.13, *p* < 0.001) and Republican party affiliation (*b* = −0.28, *p* < 0.01) negatively predicted vaccination support. Women reported lower support than men (*b* = −0.41, *p* < 0.01), and older age was negatively associated with support (*b* = −0.01, *p* < 0.001). The model accounted for 27% of the variance (*R²* = 0.27).

## Discussion

The results of the study demonstrate clear and consistent segmentation of the U.S. public based on perceived scientific source credibility. We used Latent Class analysis and identified five distinct audience segments, Estranged, Skeptics, Moderates, Optimists, and Enthusiasts, which differed significantly across credibility dimensions (competence, integrity, and goodwill), scientific optimism, and support for environmental action and health behaviors.

These findings highlight the importance of understanding segmented patterns of perceived source credibility to effectively tailor messages about environmental health topics. Perceptions of scientists’ source credibility functioned as a central differentiator among classes, with information-seeking behaviors reinforcing these patterns. For example, Enthusiasts had both the most positive perceptions of scientists’ source credibility and were more likely to rely on technical sources. This contrasted sharply with Skeptics’ and the Estranged group members’ low credibility estimates and avoidance of technical sources for information. In a politically polarizing situation, or one involving groups that are skeptical or even threatened by scientific views, simply presenting new evidence isn’t enough. In these cases, audiences may rely on ‘biased assimilation’ to more readily adopt messages that confirm their beliefs and more critically evaluate the ones that ‘disconfirm’ them (Lord et al., 1979). This can result in seeking and affirming information and evidence from sources that align with their political ideologies. Our findings reinforce previous findings of politically polarized perceptions of scientists and scientific source credibility (Gauchat, 2012; Funk et al., 2019a; Hamilton et al., 2015; Nisbet et al., 2015; Hunt & Wald 2020). For example, the Estranged class reported the lowest credibility perceptions, highest skepticism toward scientific competence, and a conservative political orientation. Their minimal institutional confidence extends beyond science to general institutions (Gauchat et al., 2017) and is associated with low engagement with scientific institutions and reduced support for environmental action. This finding supports previous scholars’ suggestion that lack of direct experiences with scientific institutions may contribute to reduced trust in scientists (Besley et al., 2021). For Skeptics, their lower confidence in scientists’ motivations and negative perceptions of scientists’ source credibility coincided with a conservation political orientation and reduced reliance on technical sources. These findings suggest that these groups may be more likely to ignore technical sources or reject scientific recommendations through biased assimilation processes (Lord et al., 1979; Nisbet et al., 2015; Pechar et al., 2018). Given the implications of these findings for biased responses to scientific communication, future research should investigate alternative sources that might be effective in engaging these groups regarding environmental and health behaviors.

To assess political polarization around issue framing, we compared two message contexts: a controversial topic (heatwaves) and a non-controversial one (salmonella). The story topic substantially shaped class configurations and attitudes, with salmonella producing different attitudinal and perception patterns than heatwaves (Beall et al., 2017; Vraga et al., 2018). This context-dependency manifested in class-specific policy support attitudes: Optimists expressed stronger support than Enthusiasts for vaccination in the salmonella context but weaker support for environmental action in the heatwave context. Moderates aligned with Enthusiasts on vaccination support but diverged sharply on environmental behaviors. Optimists’ context-dependent attitudes – exhibiting higher scientific optimism in the salmonella condition but lower support in the heatwave context – reinforce the idea that attitudes about scientists and perceived scientific source credibility are conditional, relational and likely to vary across policies and issue framing (Cologna et al., 2022; Hunt & Wald 2020). Even the Estranged class – most disconnected from science – demonstrated substantially higher confidence in science when exposed to a story about salmonella than when the topic was heatwaves. Indeed, this pattern emerged across all audience segments in this research. This finding reinforces previous findings that differential responses to scientific communication (Vraga et al., 2018), scientific legitimacy (Gauchat, 2012; Gauchat et al., 2017), and scientific goodwill (Hunt & Wald, 2018, 2020) emerge and operate differently in polarized contexts.

The model predicting science skepticism was not robust in this study. This finding has many possible drivers. Among the possibilities is the fact that this study did not measure the role of religious beliefs in shaping scientific skepticism, which has been identified by others as an important variable for understanding scientific skepticism within the U.S. population (Chinn et al., 2024). While this variable may or may not correlate with other sociodemographic factors across global regions, it warrants future investigation. It is also possible that our results derive from our focus on skeptics’ attitudes toward environmental behavior and vaccinations rather than examining direct responses to scientific recommendations. The threshold between absolute acceptance and outright rejection likely exists along a continuum rather than as a dichotomous choice. For Skeptics and the Estranged groups, support for environmental action and health behaviors may emerge through gradual attitudinal shifts from rejection toward acceptance, rather than through direct adoption or immediate support. Future research should examine these incremental transitions to better understand pathways for engaging science-resistant audiences (Fjaeran & Aven, 2021). Similarly, the Moderates class requires further investigation, as this group exhibited dramatically different patterns between contexts, mirroring Enthusiasts for the *salmonella* context and aligning more closely with skeptics regarding heatwave-related behaviors. Their reliance on technical and personal information sources, coupled with context-dependent credibility assessments, suggests susceptibility to biased assimilation depending on issue salience and framing (Featherstone et al., 2009; Lord et al., 1979; Scheufele, 2002).

It is important to note that even among groups who were generally skeptical of scientists, not all measures of source credibility were low. Many items within competence were high, even among the Estranged group. Among all classes, the lowest scored items were related to scientists’ willingness to share information, their unwillingness to welcome feedback, not reporting findings, and their failure to keep promises. These have not historically been included as key components of scientific source credibility or trustworthiness, though recent measures have included some of these items (Besley et al., 2021; Reif et al., 2025). While there has been a great deal of public discussion about the loss of public confidence in scientific expertise, we observed that competence scores were comparatively higher across audience segments than perceptions of integrity and goodwill, suggesting that decreasing public confidence in science may center more on scientists’ motivations and relational qualities than on expertise per se. These patterns underscore the importance of scientific communication that addresses not only technical knowledge but also institutional trustworthiness and genuine concern for public welfare (Cologna et al., 2025; Funk, 2020). Relatedly, in many of our models, perceived scientific motivation emerged as a critical antecedent. This aligns with previous work suggesting that the public perceives scientists as more credible when they are motivated by others’ needs rather than their own self-interest (Beall et al., 2017; Hunt & Wald, 2020). Yet previous research suggests that many scientists prioritize communication goals such as “helping to inform people about scientific issues” and “defending science from those who spread falsehoods” over “showing that the scientific community cares about society’s well-being” (Yuan et al., 2019., p. 112). This work suggests that there is a need to examine how and when scientists can demonstrate other-oriented motivations and if this approach can increase public perceptions of scientific source credibility.

Most environmental health training programs emphasize outward communication to the public rather than preparing scientists to creatively, deliberately, and meaningfully engage with their diverse audience segments to co-create trust-based coalitions (Binder et al., 2022). Few trainings to date currently prepare environmental health specialists to use bidirectional, dialogic communication and engagement strategies (Reif et al., 2025). Environmental health literacy campaigns have demonstrated that perceptions of source credibility affect message response (Binder et al., 2022). This study reinforces those findings and suggests that credibility varies among groups, across contexts and shapes attitudes toward environmental action and health behavior. For example, Hispanic/Latino respondents demonstrated significantly higher support for vaccination compared to other demographic groups in our models. Future investigations should systematically explore the mechanisms underlying this pattern, including prior exposure to large-scale vaccination campaigns, institutional trust in public health authorities, reliance on specific information sources, and perceived motivations of the scientific community among Hispanic/Latino populations. Future research could also examine how credibility emerges and can be established through dialogical public engagement across multiple environmental health contexts and social groups.

Identifying five distinct audience segments underscores the importance of tailored engagement strategies, as these groups differ fundamentally in their perceptions of scientific source credibility—especially competence, integrity, and goodwill—which serve as cognitive heuristics guiding how scientific messages are interpreted and accepted (Hovland & Weiss, 1951; McCroskey & Teven, 1999; Pornpitakpan, 2004). Enthusiasts show high institutional trust, strong perceptions of scientists’ competence and goodwill, and pro-science orientations mirror prior findings linking credibility to education and liberal ideology (Besley, 2015; Gauchat, 2011; Hayat et al., 2019). Enthusiasts are best engaged through strategies that reinforce accountability, sustain relationships with scientific institutions, and mobilize them as advocates within their networks. Optimists on the other hand exhibit generally favorable yet context-sensitive credibility judgments and benefit-focused acceptance of science (Besley, 2015; Cologna et al., 2022; Nisbet & Markowitz, 2014). We suggest that campaigns engage optimists, use consistent and coherent narratives across issues that maintain optimism while transparently addressing uncertainty to avoid perceived inconsistency or erosion of trust. Moderates’ intermediate credibility perceptions and strong susceptibility to framing effects suggest that engagement should focus on reducing polarization, clarifying the distinction between scientific uncertainty and political conflict, and leveraging cross-cutting identities to counter biased assimilation and maintain engagement (Lord et al., 1979; Hamilton et al., 2015; Li & See-To, 2023). In contrast, Skeptics and the Estranged are characterized by low institutional trust, diminished perceptions of scientific goodwill, and greater ideological resistance to expert authority (Gauchat, 2012; Hunt & Wald, 2020; Nisbet et al., 2015). This finding aligns with previous work by Nisbet and Markowitz (2014), who drawing on the theory of deference to scientific authority, suggest that optimistic and pessimistic beliefs shape ideas about science and scientific influence in society (Brossard & Nisbet, 2007). Drawing on this work and our own findings, we posit that Skeptics and Estranged group members are unlikely to respond to institution-centered or technical messaging. Instead, communication efforts are more likely to be effective when presented by trusted intermediaries, community-based or value-congruent messengers, and non-technical, experiential narratives that foreground goodwill over competence and acknowledge historical and social sources of distrust (Earle, 2012; Vraga et al., 2018; Cologna & Siegrist, 2020).

Like all research, this study has some limitations that warrant consideration. One methodological limitation is the unequal sample sizes across class segments, which may have reduced statistical power for detecting certain effects, particularly in smaller groups like the Estranged. Future research should employ purposive sampling strategies to target these classes. Although the sample was nationally representative, small cell sizes for different gender, racial, and ethnic groups prevented meaningful analysis of how gender, racial, and ethnic identity and proximity to science shape credibility perceptions. For example, due to statistical considerations we had to drop the six non-binary and transgender participants. Future studies could be designed to better represent marginalized generational, sexual orientations, gender, ethnic and racial groups such as through purposive sampling as relational credibility is essential to trust-based coalition building and collective action. The sample’s older demographic profile, with an average age around 50 years, may also limit generalizability to younger populations. Future studies could also examine urban, suburban, and rural residency effects on distance to scientists and reliance on technical information sources. Lastly, the subsampling from the larger population could have influenced representativeness and generalizability. The subsample was still randomly assigned to their conditions and still came from the same general population of US adults; therefore, we think that this choice is unlikely to affect the representational implications of the technoscientific credibility audience segmentation.

Longitudinal designs would be particularly valuable for exploring how credibility perceptions evolve over time and across different environmental health topics, especially in response to major events or misinformation campaigns, and for assessing causal relationships between credibility, beliefs, and behaviors. Finally, the digital news article used here should be tested with other populations to establish external validity of the measures and confirm the audience segmentation patterns we identified across different communication contexts. Additionally, while textual hedging in news articles did not produce significant effects in this study, further investigation is warranted, as hedging may differentially influence audience segments in spoken news announcements or other communication modalities (Hyland, 1996, 2017).

Despite these limitations, we agree with other scholars that audience segmentation based on source credibility perceptions offers a promising avenue for fostering trust-based coalition building across polarizing issues (Nisbet & Markowitz, 2014). By identifying distinct information-seeking behaviors associated with each audience segment, scientists and communicators can develop more credible messages and epistemic trust with diverse publics spanning contexts. Still, Two forces shape how people engage with science online: algorithmic personalization, which deepens polarization, and the digital divide, which stems from unequal access and literacy. Together, they hinder inclusive science communication. Future research should explore strategies that reduce polarization and bridge structural gaps, ensuring tech-based trust networks promote equity.

## Data Availability

The datasets generated during and/or analyzed during the current study are available in the Virginia Tech repository, https://figshare.com/s/95f3992f8e34417949dc.
